# Molecular characterization of HLA class II binding to the LAG‐3 T cell co‐inhibitory receptor

**DOI:** 10.1002/eji.202048753

**Published:** 2020-10-09

**Authors:** Bruce J. MacLachlan, Georgina H. Mason, Alexander Greenshields‐Watson, Frederic Triebel, Awen Gallimore, David K. Cole, Andrew Godkin

**Affiliations:** ^1^ Division of Infection & Immunity Cardiff University Cardiff UK; ^2^ Immutep S.A.S. Orsay France

**Keywords:** cancer immunotherapy, immune checkpoint inhibitors, LAG‐3, pHLA‐II, T cells

## Abstract

Immune checkpoint inhibitors (antibodies that block the T cell co‐inhibitory receptors PD‐1/PD‐L1 or CTLA‐4) have revolutionized the treatment of some forms of cancer. Importantly, combination approaches using drugs that target both pathways have been shown to boost the efficacy of such treatments. Subsequently, several other T cell inhibitory receptors have been identified for the development of novel immune checkpoint inhibitors. Included in this list is the co‐inhibitory receptor lymphocyte activation gene‐3 (LAG‐3), which is upregulated on T cells extracted from tumor sites that have suppressive or exhausted phenotypes. However, the molecular rules that govern the function of LAG‐3 are still not understood. Using surface plasmon resonance combined with a novel bead‐based assay (AlphaScreen^TM^), we demonstrate that LAG‐3 can directly and specifically interact with intact human leukocyte antigen class II (HLA‐II) heterodimers. Unlike the homologue CD4, which has an immeasurably weak affinity using these biophysical approaches, LAG‐3 binds with low micromolar affinity. We further validated the interaction at the cell surface by staining LAG‐3^+^ cells with pHLA‐II‐multimers. These data provide new insights into the mechanism by which LAG‐3 initiates T cell inhibition.

## Graphical abstract

We demonstrate that human LAG‐3 binds directly to peptide‐human leukocyte antigen class II complex (pHLA‐II) with low micromolar affinity. These findings provide new insights into the mechanism by which LAG‐3 initiates T cell inhibition and has implication for the development of novel immune checkpoint inhibitors for cancer immunotherapy.

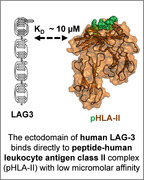

## Introduction

Immune checkpoint inhibitors (ICIs; antibodies that block T cell co‐inhibitory receptors such as PD‐1 and CTLA‐4) have revolutionized the treatment of some cancers [1, 2, 3]. Combinations of these ICIs targeting multiple pathways have demonstrated additional efficacy in certain situations, [4], showing that these co‐inhibitory receptors can act through different mechanisms.

Lymphocyte activation gene‐3 (LAG‐3) is a single pass transmembrane protein expressed on a proportion of different types of immune cells including; αβ T cells, γδ T cells, NK cells, and plasmacytoid dendritic cells. LAG‐3 is upregulated upon activation of T cells and appears to act as a co‐inhibitory receptor [5, 6]. LAG‐3 mediated suppression of T cell signaling has been shown to be dependent on the three intracellular regions in the cytoplasmic tail [7], although the signaling pathway remains unknown. Localization of LAG‐3 to the immune synapse is thought to be mediated by an interaction with peptide‐human leukocyte antigens (pHLA‐II) [8]. Indeed, LAG‐3 binds preferentially to a subset of HLA‐II molecules expressed on membrane rafts on human immature DC [9, 10] and selectively recognizes stable complexes of peptide and HLA‐II [11]. LAG‐3 expression has been implicated in maintaining the function of Treg subsets such as classical Foxp3^+^ Tregs [12]. In addition, LAG‐3 expression, in conjunction with CD49b, identifies a population of highly suppressive Foxp3^‐^ regulatory cells termed type 1 regulatory (Tr1) cells [13]. These Tr1 cells were present in tumor infiltrating lymphocytes (TILs) extracted from human colorectal cancer patients [14] and from liver tumors [15]. Together, these studies suggest LAG‐3 expression may be associated with a sub‐optimal immune response to tumors, either through direct suppression of activated effector responses or *via* the inhibitory effects of Treg populations.

**Figure 1:**
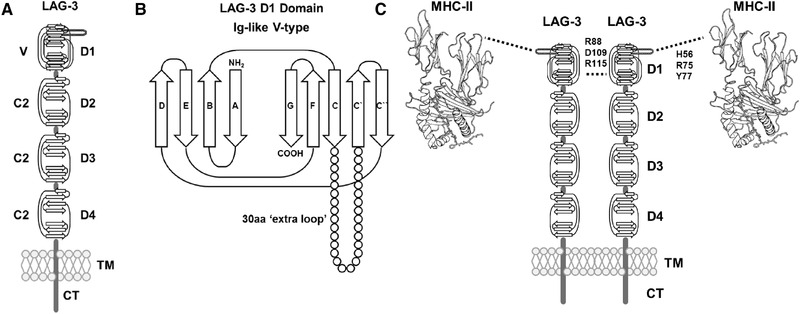
(A) Domain arrangement as inferred from the LAG‐3 protein sequence. Sequence analysis suggests LAG‐3 possesses four extracellular Ig‐like domains (D1‐D4), a single transmembrane domain (TM), and a short cytoplasmic tail (CT). D1 domain contains a V‐type Ig‐like domain (V) while D2 to D4 contains C2‐type Ig‐like domains. (B) 2D schematic representation of LAG‐3 D1 domain Ig‐like sequence inferred domain organization. The V‐type domain contains an additional 30 amino acid (aa) “extra loop” sequence between C and C`β‐strands not characteristic of V‐type Ig‐like domains. NH_2_=N‐terminus, COOH=C‐terminus. (C) Schematic overview of the hypothetic model of LAG‐3 oligomerization and pHLA‐II binding. TM, transmembrane domain; CT, cytoplasmic tail domain

Despite its well‐established role in T cell regulation, little is known about mechanisms by which LAG‐3 mediates its biological function. Although LAG‐3 was first hypothesized to bind to pHLA‐II molecules in 1990 [16], a direct interaction between the two molecules has not been formally demonstrated in the absence of any other cell surface molecule interactions. Indeed, the idea that LAG‐3 bound to pHLA‐II was first introduced because of the sequence homology between CD4 and LAG‐3, suggesting motifs characteristic of four Ig‐like domain containing proteins (Fig. 1). CD4 is an extremely potent modulator of the immune response yet has an affinity for HLA‐II that is 100–1000‐fold lower than described for other cell surface interacting T cell proteins [17]. Hence, further understanding LAG‐3 function entails understanding the binding of LAG‐3 to HLA‐II. Interactions between LAG‐3 and pHLA‐II have so far been limited to cellular studies using pHLA‐II proficient and deficient cell lines, or with pHLA‐II blocking antibodies [8, 18, 19]. Although informative, it is difficult to rule out the contributions of other molecules at the cell surface in shaping these interactions [20, 21]. Further experimental evidence, showing that LAG‐3 can block the pHLA‐II‐CD4 interaction, suggested that LAG‐3 might bind to pHLA‐II at a similar site to CD4 [22, 23], analogous to the characteristics of Ig‐like transcript 2 (ILT2) and CD8 that compete for binding to pHLA‐I [24, 25, 26].

Here, we characterized the direct interaction between LAG‐3 and pHLA‐II using purified soluble proteins. We used a novel biophysical technique (AlphaScreen^TM^) [27] as well as surface plasmon resonance (SPR) to characterize the 1:1 binding affinity of the interaction, and further demonstrated the interaction between LAG‐3 and pHLA‐II by flow cytometry by staining stable overexpressing JRT T3.5 Jurkat (JRT) LAG‐3^+^ cells with pHLA‐II multimers. Our findings confirm that LAG‐3 binds directly to pHLA‐II and suggests that this binding is independent of the HLA‐II allele or the presented peptide. The binding affinity measurements have interesting implications for the mechanism of action of this important T cell co‐inhibitory receptor. Finally, these data add to our understanding of LAG‐3 biology and will help to guide future therapeutic approaches that target this molecule.

## Results

### Direct LAG‐3:Fc binding to pHLA‐II detected by AlphaScreen^TM^

**Figure 2:**
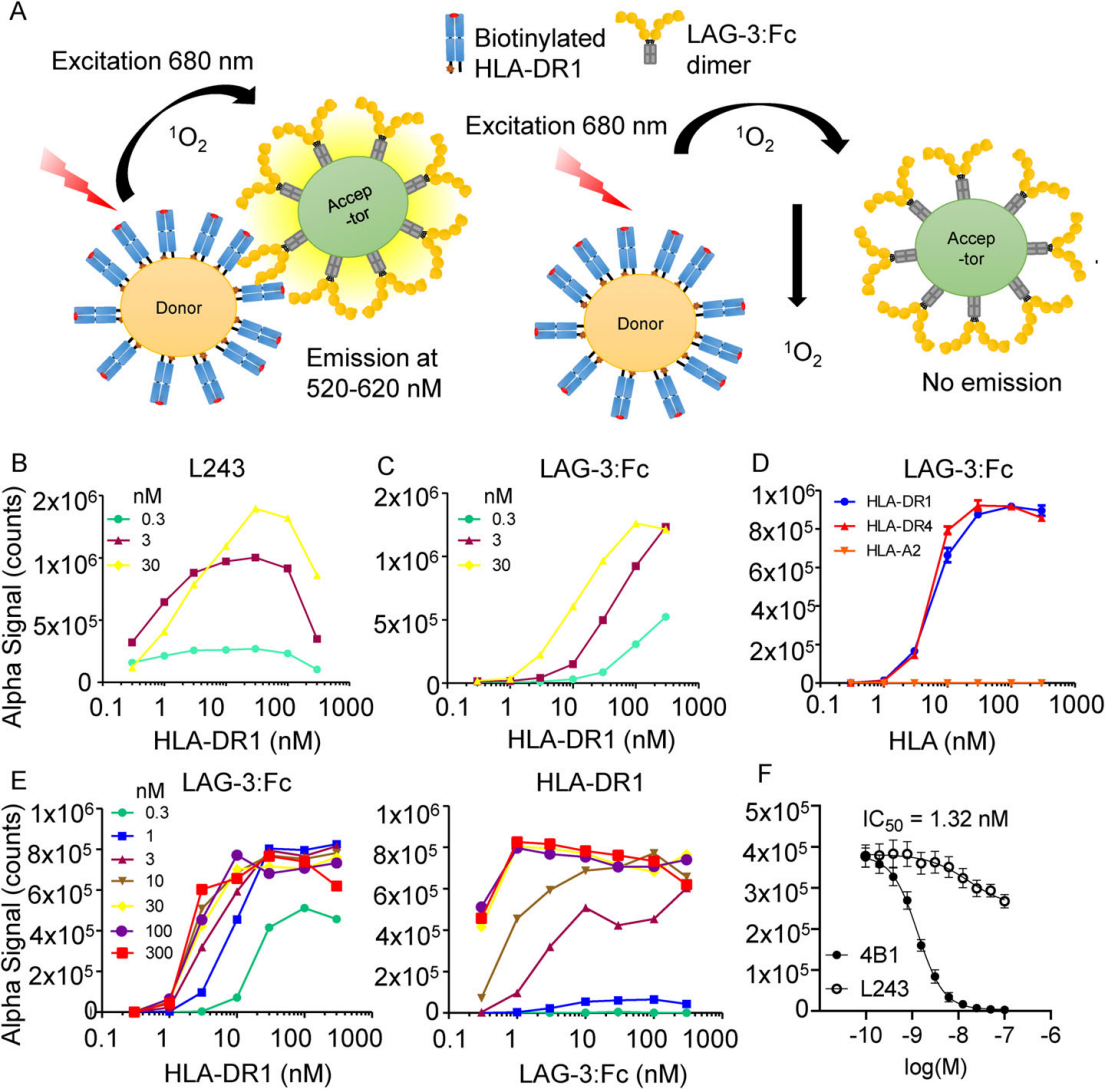
(A) Schematic overview of experimental setup for AlphaScreen^TM^ assays. (B) L243 binding to pHLA‐DR1. Assays used three concentrations of L243 antibody (0.3, 3, and 30 nM), titrated against a one in three dilution series (0.3 to 300 nM) of pHLA‐DR1. Data are representative of three independent experimental repeats using freshly prepared beads. (C) LAG‐3:Fc binding to pHLA‐DR1. Assays used three concentrations of LAG‐3:Fc (0.3, 3, and 30 nM), titrated against a one in three dilution series (0.3 to 300 nM) of pHLA‐DR1. Data are representative of three independent experimental repeats using freshly prepared beads. (D) LAG‐3:Fc binding to pHLA‐DR1 and ‐DR4. Titration of pHLA‐DR1 and ‐DR4 in one in three dilution series (300 nM to 0.3 nM). Titration of biotinylated HLA‐A*02:01 (complexed with the hTERT_540‐548_ peptide) in a one in three dilution series (300 nM to 0.3 nM) was used as a negative control. Data are representative of three experimental repeats using freshly prepared beads. (E) Cross titrations using LAG‐3:Fc and pHLA‐DR1 using a one in three dilution series (300 nM to 0.3 nM). Data are representative of three biological repeats. (F) AlphaScreen^TM^ LAG‐3:Fc/pHLA‐DR1 blockade assay using anti‐LAG‐3 4B1 and anti‐DR L243 fab fragments, IC_50_ = 1.32 nM. Data are inclusive of three independent experimental repeats using freshly prepared beads, error bars represent mean ± SEM.

Soluble LAG‐3 was generated as a LAG‐3:Fc fusion protein, expressed in glycosylation‐sufficient Chinese hamster ovary (CHO) cells to form a functionally viable and stable protein dimer as previously reported [19]. This dimer of LAG‐3 has been used extensively to explore LAG‐3 function as a therapeutic agent [28]. Efforts were also made to generate a monomeric form of LAG‐3, but without success. In order to test the interaction between LAG‐3:Fc and pHLA‐II, we produced soluble HLA‐DRA1*01:01/HLA‐DRB*01:01 (HLA‐DR1), and HLA‐DRA1*01:01/HLA‐DRB*04:01 (HLA‐DR4) using previously published methodology [29, 30]. We initially chose a highly sensitive screening method, Amplified Luminescent Proximity Homogeneous Assay Screen (AlphaScreen^TM^) [27], to test the interaction between the LAG‐3:Fc and pHLA‐II proteins [31]. AlphaScreen^TM^ is a bead‐based protein–protein interaction detection assay in which, upon excitation of a donor bead with low energy red‐shifted light (680 nm), the photosensitizing phthalocyanines in the bead release electronically excited singlet oxygen (^1^O_2_) molecules (Fig. 2A). Singlet oxygen molecules can diffuse in solution up to 200 nm (higher than 1−10 nm achieved with Forster resonance energy transfer) due to their limited half‐life. This results in a cascade of anthracene rubrene and subsequent emission of light at 520−620 nm. A single donor bead releases up to 60 000 singlet oxygen molecules, so the signal of one biological molecule to this bead is highly amplified. This results in high sensitivity and a dynamic range with very low background signal.

We first validated the AlphaScreen^TM^ assay using pHLA‐DR1 and L243 (a well‐characterized HLA‐DR‐specific antibody that binds to conformationally intact heterodimers [32, 33]). AlphaScreen^TM^ signal was detected in a concentration dependent manner using a cross titration of pHLA‐DR1 on donor beads, and L243 on acceptor beads (Fig. 2B). AlphaScreen^TM^ signal was also detected in a concentration dependent manner using a cross titration of either pHLA‐DR1 or pHLA‐DR4 on donor beads, and LAG‐3:Fc on acceptor beads (Fig. 2C and D). No binding was detected when pHLA‐A*02:01 (pHLA‐A2) donor beads were used (Fig. 2D). We confirmed these data using a larger range of acceptor bead protein concentrations (Fig. 2E; Supporting Information Fig. S1). Of note, binding was detected even at low LAG‐3 concentrations (0.3 nM), implying that the interaction might be of relatively strong affinity. We also attempted to measure the interaction between pHLA‐DR1 and CD4 but, consistent with the very weak published affinity [17], we did not detect a signal (data not shown). Finally, we demonstrated that the AlphaScreen^TM^ signal detected through LAG‐3:Fc binding to pHLA‐DR1 could be blocked using a LAG‐3 specific antibody (Fig. 2F).

### LAG‐3 binds to pHLA‐DR1 independently of the peptide with low micromolar affinity

**Figure 3:**
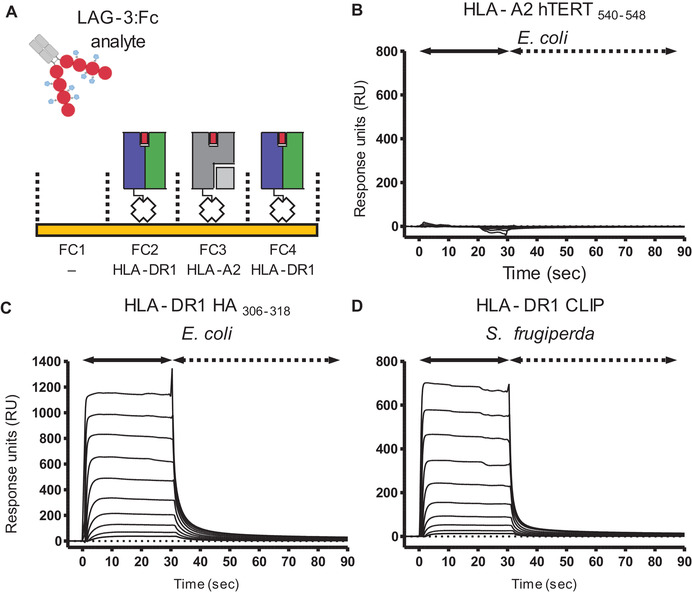
(A) Cartoon schematic describing formulation of SPR experiments performed using LAG‐3:Fc as analyte injected over pHLA immobilized sensor chips. FC, flow cell. (B) SPR analysis of LAG‐3:Fc injection over immobilized HLA‐A*02:01 complexed with the hTERT_540‐548_ peptide. Sensograms reference subtracted from a blank no‐ligand control flow cell. Data are representative of two independent experimental repeats (*n* = 2). (C) SPR analysis showing concentration‐dependent binding (57 μM to 0.11 μM) of LAG‐3:Fc to HLA‐DR1 produced from *E. coli* by in vitro refolding in the presence of the HA_306‐318_ peptide. Data are representative of two independent experimental repeats (*n* = 2). (D) SPR analysis showing concentration‐dependent binding (57 μM to 0.11 μM) of LAG‐3:Fc to HLA‐DR1 covalently‐linked to CLIP and produced in *sf9* insect cells. Data are representative of two independent experimental repeats (*n* = 2).

SPR analysis, using streptavidin immobilized pHLA‐II and LAG‐3:Fc as the analyte (Fig. 3A), demonstrated specific (Fig. 3B), concentration‐dependent binding to HLA‐DR1 complexed with the HA_309‐318_ peptide (Fig. 3C) and binding was not altered by the expression system used to generate pHLA‐DR1 or the presented peptide (as shown by equal binding to pHLA‐DR1 produced via a covalently linked CLIP construct in *sf9* insect cells) (Fig. 3D). The interaction was characterized by rapid binding kinetics (k_on_ and k_off_) in line with previously published interactions between HLA molecules and both TCRs and co‐receptor molecules [34].

To determine the monovalent affinity between LAG‐3 and pHLA‐II, we first explored the valency exhibited by the dimeric LAG‐3:Fc fusion protein. To this end, various simultaneous k_on_/k_off_ modeling curves were applied to observed binding sensograms at LAG‐3:Fc concentrations of 0.1–7 μM and compared for visual goodness‐of‐fit as well as analysis of *χ* ^2^ and residual plot parameters (Supporting Information Fig. S2A). No model demonstrated perfect matching to observed data across multiple concentrations. However, kinetic data were best described by the bivalent analyte model of binding by satisfying multiple analyzed analyte concentrations. Bivalent analytes can exhibit altered kinetics at different analyte concentrations making fitting global parameters that satisfy multiple protein injection concentrations difficult. Analysis of kinetic fitting to a single intermediate concentration of analyte (local fitting) resulted in a similar good fit and comparable kinetic parameters to those observed in global fitting (Supporting Information Fig. S2B). While LAG‐3:Fc binding was best described by the bivalent analyte model, it was observed that dissociation of LAG‐3:Fc unexpectedly occurred with fast kinetics; within the timescale of seconds. Such fast kinetics are atypical of dimeric molecules binding with bivalency. To determine the rates of monovalent and bivalent interactions to the overall observed kinetics, the kinetic parameters of bivalent binding (*k_on1_*, *k_off1_*, *k_on2_*, and *k_off2_*) were calculated from kinetic fits (Table 1). From this analysis, the affinity constant K_D1_, which defines the monovalent component of the interaction was calculated. Analysis of the kinetic derived equilibrium dissociation constants suggested that the contribution of bivalent effects to observed LAG‐3:Fc binding was minimal, as signified by a higher calculated dissociation constant for the bivalent component (K_D2_) and fast off‐rates (Table 1). As a result, the observed kinetics were dominated by 1:1 binding with minimal contribution of bivalency.

**Table 1:** Bivalent analyte model parameters of LAG‐3:Fc binding to HLA‐DR1

Fit	[Analyte] (μM)	R_max_ (RU)	RI (RU)	*χ* ^2^	k_on1_ (M^−1^ s^−1^)	k_off1_ (s^−1^)	k_on2_ (M^−1^ s^−1^)	k_off2_ (s^−1^)	K_D1_ (M)	K_D2_ (M)	K_D1_ (μM)
Local	7.1	1190	6.6	39.6	7.24 × 10^4^	0.439	156.6	4.30 × 10^−3^	6.06 × 10^−6^	2.75 × 10^−5^	6.06
Global	0.1 – 7.1	962	4.6 – 16.9	27.6	5.22 × 10^4^	0.380	96.9	4.10 × 10^−3^	7.28 × 10^−6^	4.23 × 10^−5^	7.28

[Analyte] = Concentration of injected LAG‐3:Fc analyte, RU = Response units,

R_max_ = Maximum analyte binding capacity in RU, RI = Bulk refractive index effect in RU,

$\chi 2=\sum \frac{{\left(observed-expected\right)}^{2}}{expected};$ lower χ^2^ values indicate better model fitting.

*K_on2_ was converted to M^−1^ s^−1^ via the following equation: k_on2_ (M^−1^ s^−1^) = k_on2_ (RU^−1^ s^−1^) × 100 × molecular weight of LAG‐3:Fc [44, 45].

K_D1_= the affinity equilibrium dissociation constant (monovalent component).

K_D2_ = the affinity equilibrium dissociation constant (bivalent component).

In order to deduce the monovalent affinity for LAG‐3:Fc binding to pHLA‐DR1, we attempted to remove the weak component of bivalent binding by lowering the density of ligand on the chip surface. We also attempted to immobilize LAG‐3:Fc on the chip surface, but without success. Lower ligand density increases the spatial distance between molecules and, as a result, reduces the availability of a second ligand species to engage with bivalency. The presence of bivalency can therefore also be confirmed by the increase in ligand density, increasing bivalent binding and consequently increasing observed avidity effects (Supporting Information Fig. S3A). Different concentrations of pHLA‐DR1 was immobilized to flow cells of the same sensor chip such that the effect of LAG‐3:Fc analyte binding to low, intermediate, and high ligand densities could be studied (Supporting Information Fig. S3B and C). In agreement with described kinetic modelling of LAG‐3:Fc binding, increase in ligand density prolonged binding kinetics by lengthening the observed K_off_ of LAG‐3:Fc. Similarly to previous kinetic analysis, at the highest ligand density tested, LAG‐3:Fc did not exhibit antibody‐like K_off_, again suggesting that LAG‐3:Fc did not efficiently engage with multiple pHLA‐DR1 molecules. Experiments at low ligand concentration (244 response units of pHLA‐DR1) displayed fast kinetics and no extension of dissociation. As a result, LAG‐3:Fc binding at low ligand density exhibited one‐to‐one monovalent binding behavior. Indeed, low ligand density sensograms did not adhere to a bivalent kinetic model fitting (data not shown).

**Figure 4:**
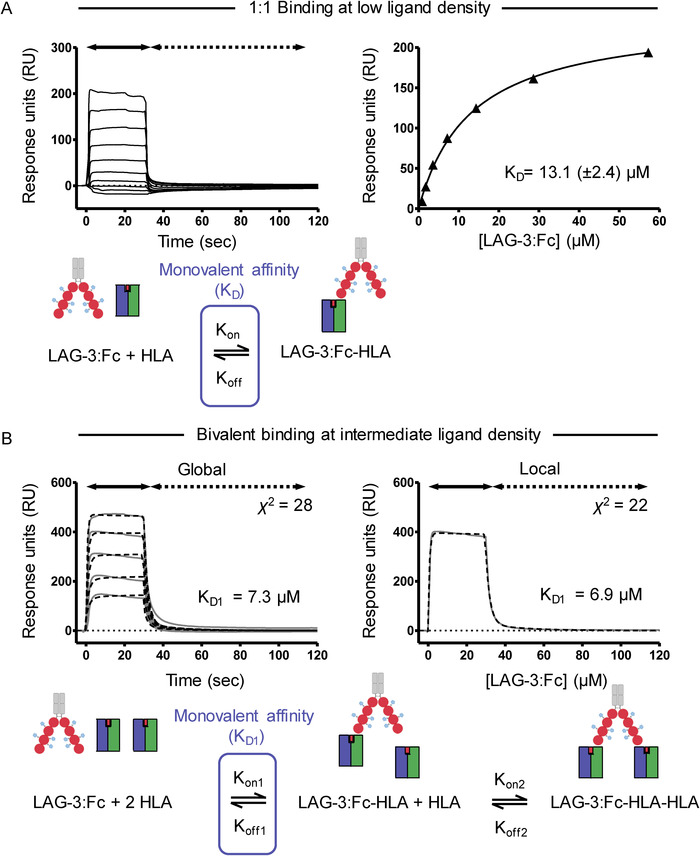
(A) Top left: SPR analysis of LAG‐3:Fc binding to pHLA‐DR1 immobilized at low (244 RU) ligand concentration. Top right: Steady‐state analysis of LAG‐3:Fc binding to pHLA‐DR1 at low ligand concentrations analyzed from sensograms shown in A by plotting RU increase from baseline during steady‐state (30 seconds into injections) against concentration of LAG‐3:Fc. Data are representative of two independent experimental repeats (*n* = 2). Bottom: Schematic representation of LAG‐3:Fc binding to a single pHLA‐DR species at low ligand density and the applied steady‐state 1:1 binding model by which a monovalent affinity (K_D_) was calculated. (B) Top left: Global fit analysis of reference subtracted sensograms of LAG‐3:Fc (0.1–7 μM) binding to immobilized (525 RU) pHLA‐DR1 fitted with a bivalent analyte model. Observed sensograms are shown as grey solid lines, fitted curves as black dashed lines with inset χ^2^ value and kinetic derived dissociation affinity constant K_D1_. Corresponding curve fit residual plots are shown below each fit. Top right: Local fit analysis of the reference subtracted sensogram describing LAG‐3:Fc binding at 7 μM to immobilized pHLA‐DR1. Data are representative of two independent experimental repeats (*n* = 2). Bottom: Schematic representation of LAG 3:Fc binding with bivalency at intermediate ligand density. The applied bivalent binding model is shown, highlighting the monovalent component of the binding model by which a monovalent affinity (*K*_D1_) was calculated.

As SPR data at low ligand density exhibited monovalent binding, a steady state derived affinity could be calculated. Using this described steady‐state analysis, the monovalent affinity of LAG‐3 binding to pHLA‐DR1 was K_D_ = 13.1 μM (Fig. 4A). Since the lowest ligand density tested may still contain contributions from bivalent binding, the calculated monovalent K_D_ value under the assumption of one‐to‐one binding may over‐calculate the monovalent affinity, i.e., the monovalent affinity might appear artificially stronger due to a remaining contribution from bivalency. In order to further validate the assumption of one‐to‐one binding, SPR data at intermediate density was analyzed by kinetic analysis. Global and local fitting of the bivalent analyte model revealed an equilibrium dissociation constant (K_D1_) for the monovalent affinity for LAG‐3 to pHLA‐DR1 within a similar range as shown by equilibrium binding analysis (K_D1_ = 6.9–7.3 μM) (Fig. 4B). This similar kinetic rate derived affinity constant validates the assumption that low ligand density steady‐state analysis was predominated by monovalent binding. Together, these data therefore define the affinity of LAG‐3 for pHLA‐DR1 loaded with the high affinity peptide HA_306‐318_ to be ∼6.9–13.1 μM at 25°C.

### pHLA‐DR1 multimers bind to LAG‐3^+^ cells at the cell surface

**Figure 5:**
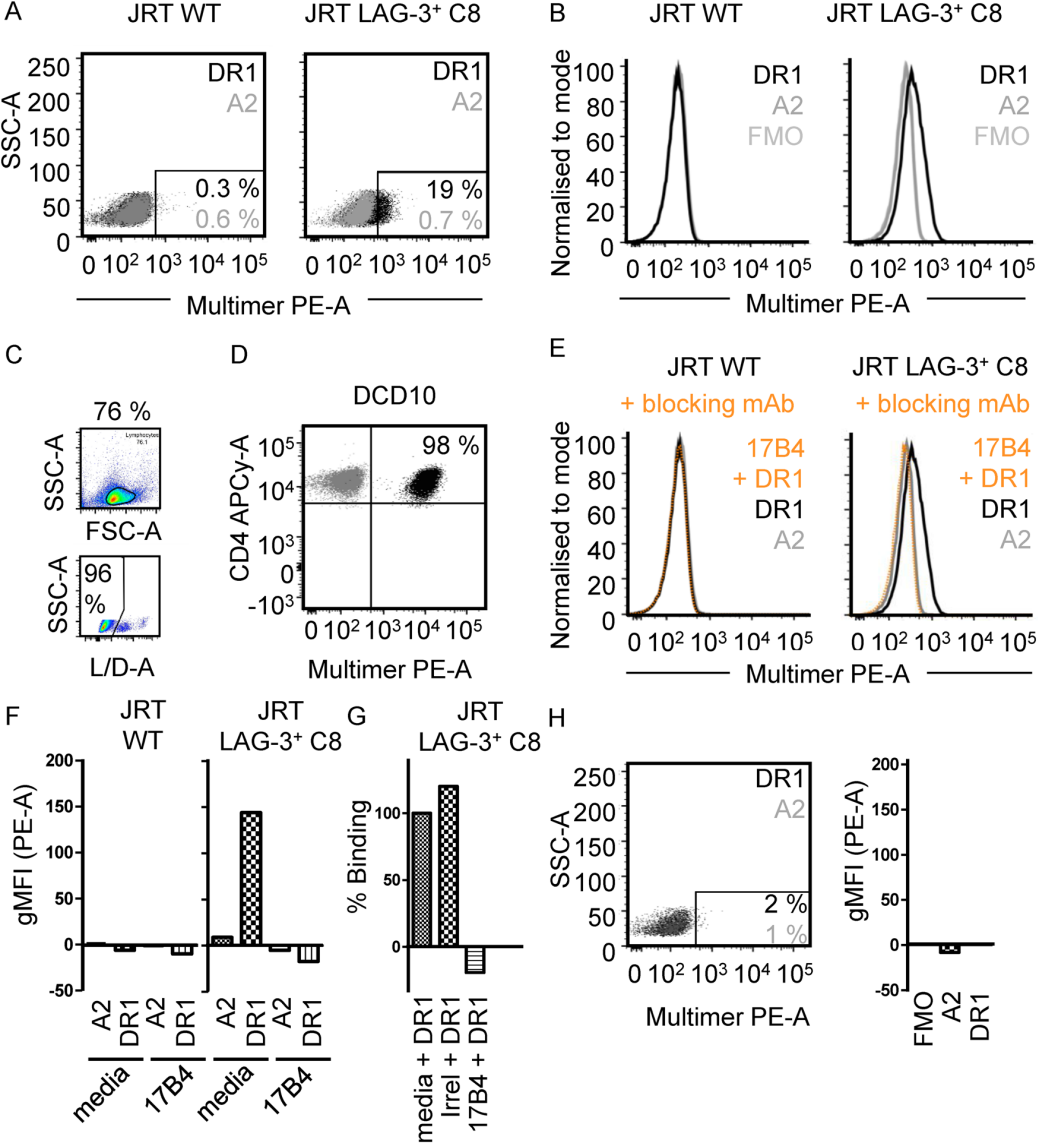
(A) 2D Flow cytometry plots of pHLA‐multimer staining of JRT WT cells (left) and JRT LAG‐3^+^ C8 cells (right) with pHLA‐A*02:01 multimers (grey) or pHLA‐DR1 multimers (black). Inset numbers = percentage multimer^+^ cells. Data are representative of three independent experimental repeats with one sample per experiment. (B) Flow cytometry histograms of pHLA‐multimer staining as in (A). Geometric mean fluorescent intensity (gMFI) values are indicated in the inset. Data are representative of three independent experimental repeats with one sample per experiment. (C) Example gating strategy of lymphocyte gate and live L/D‐A^‐^ cells used for all flow cytometry analysis. Example shown is JRT LAG‐3^+^ C8 cells stained with pHLA‐DR1 multimers. (D) 2D Flow cytometry plot of cognate CD4^+^ T cell clone (DCD10) staining with pHLA‐DR1 multimers used in experiments described. Cognate (DR1_PKY_) and irrelevant (DR1_irrel_) multimer stains are shown. Data are representative of two independent experimental repeats with one sample per experiment. (E) Flow cytometry histograms of pHLA‐multimer staining of JRT WT cells (left) and JRT LAG‐3^+^ C8 cells (right) stained with pHLA‐DR1 multimers pre‐blocked with unconjugated anti‐LAG‐3 mAb clone 17B4 (orange; dashed) or media control (black). Data are representative of two independent experimental repeats with one sample per experiment. Data shown in (B) and (E) were performed as a single experiment and are depicted separately for clarity. (F) FMO subtracted quantification of gMFI in experiments described in (E). Data are representative of two independent experimental repeats with one sample per experiment. (G) Quantification of pHLA‐DR1 binding to JRT LAG‐3^+^ C8 cells pre‐incubated with control (media), irrelevant mAb (irrelevant; anti‐CD4) or anti‐LAG‐3 mAb (17B4). Percentage binding normalized to background subtracted gMFI observed for control blockade of pHLA‐DR1 binding. Data are representative of two independent experimental repeats with one sample per experiment. (H) Left: 2D Flow cytometry plots of pHLA‐II‐multimer staining of LAG‐3^‐^ CD4^+^ MOLT‐3 cells with pHLA‐A*02:01 multimers (grey), or pHLA‐DR1 multimers (black). Inset numbers = percentage multimer^+^ cells, color coded as described. Data are representative of two experimental repeats with one sample per experiment. Right: Staining of LAG‐3^‐^ CD4^+^ MOLT‐3 cells with either pHLA‐A*02:01 or pHLA‐DR1 multimers. Data are a representative of two experimental repeats with one sample per experiment. All data in graphs are single gMFI values from a representative example experiment.

In order to confirm the molecular interaction of LAG‐3 and pHLA‐II on the surface of LAG‐3 expressing cells, a stably expressing LAG‐3^+^ JRT clone (JRT LAG‐3^+^ C8; derived from J.RT3‐T3.5 Jurkat cells) was produced (Supporting Information Fig. S4A and B). JRT WT, JRT LAG‐3^+^ C8 cells, and the MOLT‐3 cell line (an LAG‐3^‐^ CD4^+^ control) were analyzed for TCR, LAG‐3, and CD4 expression demonstrating LAG‐3 expression only on JRT LAG‐3^+^ C8 cells and CD4 expression only on MOLT‐3 cells (Supporting Information Fig. S5). JRT WT and JRT LAG‐3^+^ C8 cells were stained using PE labeled pHLA‐DR1 dextramers using a protocol optimized for low affinity interactions [35]. Staining of JRT LAG‐3^+^ C8 cells with HLA‐DR1 multimers loaded with HA_306‐318_ peptide resulted in a modest detection of LAG‐3 replete cells compared to both FMO and pHLA‐A2 multimer controls, while JRT WT cells exhibited no staining (Fig. 5A and B). Staining of LAG‐3 replete cells with pHLA‐DR1 multimers resulted in 19% of cells exhibiting detectable staining compared to background levels. To ensure the observed multimer staining did not arise due to multimer deposition on dead cells, all experiments described were gated on live lymphocyte cells by live/dead staining (Fig. 5C). Despite the observed staining of JRT LAG‐3^+^ C8 cells by pHLA‐DR1 multimers, such staining was poor compared to staining of a previously published HLA‐DR1‐HA_306‐318_‐specific cognate T cell clone (DC‐C10) [36], where 99% of cells were detected as multimer^+^ (Fig. 5D). In order to confirm that the observed staining of LAG‐3^+^ cells by pHLA‐DR1 multimers was mediated by LAG‐3, the effect of the LAG‐3 blocking antibody, clone 17B4, on pHLA‐DR1 multimer staining was assayed. Indeed, pre‐incubation of JRT LAG‐3^+^ C8 cells before multimer staining abrogated the shift in fluorescence intensity observed when staining with pHLA‐DR1 multimers (Fig. 5E). To confirm antibody blockade had no effect on pHLA‐A2 multimer staining, corresponding blockade experiments were also performed on such controls and resulted in no significant impact on background staining (Fig. 5F). Moreover, an irrelevant antibody (αCD4) was not able to abrogate the binding of pHLA‐DR1 multimers to JRT LAG‐3^+^ C8 cells (Fig. 5G). Since CD4 also binds to pHLA‐II molecules, albeit via a very weak interaction [17], it was reasoned that a similar staining of CD4^+^ cells by pHLA‐II multimers may be observed. The CD4^+^ LAG‐3^‐^ MOLT‐3 cell line, however, showed no significant detection of multimer^+^ cells using pHLA‐DR1 multimers (Fig. 5H).

## Discussion

LAG‐3 is a target for the next wave of checkpoint immunotherapies, but little is known about the biology of its ligands, and how this relates to its function. Previous evidence has suggested pHLA‐II as a receptor for LAG‐3 [8, 16, 18, 19, 22, 23], but the cell‐adhesion molecule LSECtin/CLEC4G [21], Galectin‐3 [37], α‐synuclein [38], and fibrinogen‐like protein‐1 [20], have also been proposed as LAG‐3 ligands. Here, we used a cutting‐edge microbead interaction assay (AlphaScreen^TM^), combined with SPR analysis and cellular flow cytometry to characterize the direct protein–protein interaction between LAG‐3 and pHLA‐II using a well characterized bivalent LAG‐3:Fc fusion protein [19]. SPR and AlphaScreen^TM^ analyses demonstrated that LAG‐3 bound independently of the HLA allele tested (HLA‐DR1 and HLA‐DR4) and the presented peptide (HA_306‐318_ and CLIP) with an affinity (K_D_) of 6.9–13.1 μM. This affinity is comparable to that of TCR engagement of pathogen‐derived peptides and is higher than average TCR‐pHLA‐II binding [30]. However, this μM affinity is several log orders of magnitude weaker than the calculated avidity of dimeric LAG‐3 binding in indirect cell surface experiments (K_D_ = 60 nM at 37°C) [19]. Recent evidence suggests that LAG‐3 might selectively bind to stable pHLA‐II complexes, but not unstable pHLA‐II complexes, via a conformationally dependent mechanism [11]. Although we did not test any unstable pHLA‐II complexes in our study (HA_306‐318_ is known to be a strong HLA‐DR1 binder, and CLIP was stabilized via a covalent linker), our data showing that LAG‐3 binding was unaffected by these two stable peptides is in line with this notion.

Despite the weak affinity, these studies show LAG‐3 binds pHLA‐DR1 at a significantly stronger affinity than the CD4 co‐receptor, for which the solution affinity has been estimated as 2.5 mM [17]. As LAG‐3 binding to pHLA‐II molecules has been shown to block consequent CD4 binding at the cell surface [19], competition between LAG‐3 and CD4 for pHLA‐II binding would favor engagement by LAG‐3, potentially contributing to LAG‐3 mediated T cell inhibition. It is noteworthy that the LAG‐3 affinity for pHLA‐II reported here (K_D_ = 6.9–13.1 μM) is similar to that of the pHLA‐I co‐inhibitory receptor, ILT2 (K_D_ = 7 μM) [26], which interferes with pHLA‐I restricted T cell function [39]. We also demonstrated that pHLA‐DR1 multimers weakly stain LAG‐3^+^ cells. Such staining could not be attributed to TCR binding due to the lack of TCR‐β chain expression in the JRT3 T3.5 Jurkat cell line as confirmed by minimal staining of JRT LAG‐3^+^ C8 cells with a pan‐αβ TCR antibody. The observed staining was instead shown to be LAG‐3 dependent by the blockade of multimer staining by the 17B4 clone αLAG‐3 antibody. Although 100% LAG‐3 expression was observed for the JRT LAG‐3^+^ C8 cells, only a fraction of cells were detected by pHLA‐DR1 multimers. Despite the relatively weak (compared to pHLA‐II staining of TCRs) staining we observed, these data have important implications for CD4^+^ T cell biology experimental design in order to prevent misdetection of LAG‐3^+^ cells as perceived antigen‐specific multimer^+^ cells.

Importantly, staining of LAG‐3^+^ cells with pHLA‐DR multimers was far weaker than the staining we observed on T cells using cognate peptide loaded pHLA‐DR multimers. This was regardless of the stronger binding affinity of the pHLA‐II‐LAG‐3 interaction compared to the TCR‐pHLA‐II interaction. As there is evidence that LAG‐3 forms dimeric molecules at the cell surface [40], these findings raise interesting questions over the role of LAG‐3 dimerization contributing to an increase in functional potency. For CD4, despite a weak affinity, pHLA‐II‐CD4 binding maintains TCR phosphorylation at an active basal level by recruitment of Lck and thus poises T cells for cognate TCR‐pHLA engagement [17]. Since LAG‐3 also exhibits fast kinetic binding to pHLA‐II, parallels between LAG‐3 and CD4 co‐receptor maintenance of antigen specificity may exist. Indeed, surface bound LAG‐3 mediated inhibition of DC maturation has been shown to be antigen specific and require TCR‐pHLA‐II engagement by LAG‐3 expressing Tregs [41]. Thus, the interaction between LAG‐3 and pHLA‐II at the cell surface may be tuned such that ligand discrimination is maintained. However, the role that LAG‐3 plays on activated human T cells is poorly understood. Consequently, further knowledge of LAG‐3 signaling is required to understand how this observed affinity for pHLA‐II affects LAG‐3 mediated T cell homeostasis, as well as considering the potential role of other LAG‐3 ligands [20, 21, 37, 38].

In summary, we show that LAG‐3 directly interacts with different HLA‐II alleles with low micromolar affinity, independently of the presented peptide, HLA‐II allele and glycosylation state of the pHLA‐II molecule. As a result, these data add evidence to the model where LAG‐3 outcompetes CD4 for pHLA‐II binding during LAG‐3 mediated T cell suppression. Our findings have interesting implications for LAG‐3 mediated T cell biology, which is a major focus for the development of novel checkpoint immunotherapies.

## Materials and methods

### Production of soluble pHLA‐DR1 and LAG‐3:Fc

Soluble pHLA‐DR1 was either refolded from recombinant DR1α and DR1β chains produced in the BL21(DE3) strain of *E. coli* [29], or generated in *Spodoptera frugiperda* (sf9) insect cells using the BaculoDirect™ expression system (ThermoFisher Scientific) [30] as previously described. pHLA‐DR1 molecules were biotinylated by inclusion of a C‐terminal AviTAG™ biotinylation signal sequence on the DR1α chain that was biotinylated using a BirA biotin‐protein ligase kit (Avidity) [36]. A fusion protein of the four extracellular domains of LAG‐3 and the Fc domain of IgG (LAG‐3:Fc) was produced in CHO cells as previously described [19]. LAG‐3:Fc was stable in a solution of 20 mM sodium citrate, 86 mM sodium chloride, 100 mM l‐arginine, 0.02% Tween‐20, pH 7.4 with citric acid (TBSB buffer) at concentrations of up to 30 mg/mL.

### AlphaScreen^TM^ analysis

Due to the nature of the AlphaScreen^TM^ bead‐based proximity assay (Perkin Elmer), optimal protein concentration that results in optimal coating of the bead is unique to each protein and bead combination. Therefore, protein cross titrations were performed. For each protein individual 4× working solutions were prepared using AlphaLISA^TM^ buffer. These were used to produce a serial dilution for each protein sample. Each dilution series also included an AlphaLISA^TM^ buffer alone in order to measure background signal. The protein dilutions (10 μL of each), were loaded into a 96‐well half area opti‐plate. The plate was sealed and incubated for 1 h at room temperature. The 4× working solution (20 μg/mL final concentration) of protein A acceptor beads and streptavidin donor beads weres prepared using AlphaLISA^TM^ buffer keeping donor beads protected from light. Equal volume of each bead solution was mixed and 20 μL of this combined bead solution was loaded into all wells containing pre‐incubated protein samples. The plate was then re‐sealed and incubated at room temperature for 1 h. The plate seal was then removed, and the plate read by an En‐vision plate reader (Perkin Elmer).

### Anti‐LAG‐3 fab fragment blocking assay

Direct blocking assays were conducted for the LAG‐3:Fc‐pHLA‐DR1‐biotinylated AlphaScreen^TM^ assay. Due to the use of protein A beads to capture LAG‐3:Fc, the antibodies (αLAG‐3 4B1 in house generated and αHLA‐DR1 L243 (Biolegend)) were first papain digested using a Pierce™ Fab Preparation Kit (ThermoFisher Scientific) according to the manufactures instruction, to remove antibody Fc fragments. To prevent the antibodies binding to the protein A beads, serial diluted fab fragments were then added to fixed concentrations of LAG‐3:Fc and pHLA‐DR1. AlphaScreen^TM^ assays were performed as described above but using 5× working solutions to account for addition of 10 μL of antibody fab fragment samples to the protein incubation step.

### Analysis of LAG‐3‐pHLA‐DR1 binding via SPR

LAG‐3:Fc sample was gel filtrated into fresh TBSB buffer the day of SPR experiments using a Superdex S200 10/300 column and ÄKTA Pure fast protein liquid chromatography system (GEHealthcare Life Sciences). Analysis of LAG‐3:Fc‐pHLA‐DR1 binding was performed on a BIAcore T200 instrument (GE Healthcare Life Sciences). All experiments were performed at 25°C in TBSB buffer. Prepared biotinylated pHLA‐DR molecules were immobilized to covalently linked streptavidin coated CM5 sensor chips prepared as previously described [42]. Biotinylated pHLA‐DR molecules were bound to the chip surface at a flow rate of 10 μL/min. LAG‐3:Fc molecules were twofold serially diluted and sequentially injected at 30 μL/min (30 s association, 300 s dissociation) over the pHLA‐DR chip surface. Recorded sensograms were reference subtracted against a control flow cell of HLAA*02:01‐hTERT_540‐548_ after confirmation as a suitable non‐binding control ligand. Sensograms were analyzed using BIAevaluation version 4.1 (GE Healthcare Life Sciences) and plotted using GraphPad Prism version 5 (GraphPad Software, Inc). Kinetic analyses of LAG‐3:Fc binding were performed using the simultaneous k_on_/k_off_ fitting function of the binding model specified. Equilibrium analyses were performed using nonlinear regression least squares ordinary fit of the one‐site specific binding model using GraphPad Prism version 5.

### Generation of LAG‐3^+^ Jurkat cells

The full length sequence of human LAG‐3 (Uniprot: P18627) flanked by a 5’ XbaI restriction site (TCTAGA), Kozak sequence (GCCGCCACC) and start codon (ATG) and a 3’ XhoI restriction site (CTCGAG) was codon optimized for human expression and cloned into the pELNsxv third generation lentiviral transfer vector (kindly provided by Prof. James Riley, University of Pennsylvania) containing a self‐cleaving P2A linked rat CD2 (rCD2) expression cassette within the multiple cloning site (P2A.rCD2.pELNsxv). LAG‐3 encoding lentiviral particles were generated in HEK 293T cells (ATCC® CRL‐3216™) by calcium chloride transfection of LAG‐3.P2A.rCD2.pELNsxv with the lentiviral packaging plasmids pMD2.G (Addgene plasmid #12259), pMDLg/pRRE (Addgene plasmid #12251), and pRSV‐Rev (Addgene plasmid #12253) that were gifts from Didier Trono [43]. J.RT3‐T3.5 Jurkat cells (JRT) (ATCC® TIB‐153™) were transduced with LAG‐3.P2A.rCD2 lentiviral supernatents by incubating 2 × 10^5^ cells seeded in a 48‐well plate and incubating overnight at 37˚C and 5% CO_2_. Transduction of JRT cells was assessed by fluorescent antibody staining of rCD2 (PE‐conjugated clone OX‐34; Biolegend) and LAG‐3 (PE‐conjugated goat polyclonal; R&D Systems) using a BD FACSCanto II flow cytometer. Data were analyzed using FlowJo (Tree Star Inc.). Transduced JRT cells were subsequently cloned using limiting dilution to obtain a clonal LAG‐3 expressing population (JRT LAG‐3^+^ C8).

### pHLA‐DR multimer staining of LAG‐3^+^ Jurkat cells

Multimer staining was performed on JRT LAG‐3^+^ C8, JRT WT, and MOLT‐3 cells (ATCC CRL‐1552^TM^). TCR (FITC‐conjugated clone IP26; BioLegend), LAG‐3 (FITC‐conjugated clone 17B4; Enzo Life Sciences), and CD4 (APCy‐conjugated clone VIT4; Miltenyi Biotec) expression by JRT LAG‐3^+^ C8, JRT WT, and MOLT‐3 cells was characterized as single stains prior to multimer staining experiments. PE‐conjugated pHLA‐DR dextramers (Immudex) were prepared as previously described [36]. Prepared multimers were used to stain JRT LAG‐3^+^ C8, JRT WT, and MOLT‐3 cells using a protocol optimized for low affinity interactions that included pretreatment of cells with the protein kinase inhibitor dasatinib and boosting staining with unconjugated anti‐PE (clone PE001; BioLegend) [35]. For antibody blocking experiments, cells were plated and washed then incubated with 10 μg/mL unconjugated anti‐LAG‐3 (clone 17B4), irrelevant control anti‐CD4 (clone VIT4), or media for 1 h on ice prior to multimer staining. Cells were stained for viability using LIVE/DEAD® Fixable Violet Dead Cell Stain (Thermo Fisher Scientific).

## Conflict of interest

D.K.C. is currently an employee of Immunocore Ltd. F.T. is currently and employee of Immuntep Ltd. The other authors declare no commercial or financial conflict of interest.

## Author contributions

B.J.M., G.H.M., A.G.W., F.T., D.K.C., and A.G. performed and/or directed experiments, analyzed data, and critiqued the manuscript. D.K.C. and A.G. conceived, funded, and directed the project. D.K.C., B.J.M., G.H.M., and A.G. wrote the manuscript.

### Peer review

The peer review history for this article is available at https://publons.com/publon/10.1002/eji.202048753.

**Abbreviations**
APCy: allophycocyanin
CHO: Chinese hamster ovary cells
EDC: N3‐ethylcarbodiimide
HA: hemaggluttinin
ILT2: Immunoglobulin‐like transcript 2
JRT: JRT T3.5 Jurkat cells
LAG‐3: Lymphocyte activation gene‐3
NHS: N‐hydroxysuccinimide
pHLA‐DR1: HLA‐DRA*0101/HLA‐DRB*01:01
pHLA‐DR4: HLA‐DRA*0101/HLA‐DRB*04:01
pHLA‐II: peptide‐human leukocyte antigen class II
RU: response units
SPR: surface plasmon resonance
TCR: T cell receptor
TILs: tumor infiltrating lymphocytes
Tr1: type 1 regulatory cells
T_regs_: FoxP3^+^ regulatory T cells

## Supporting information

Supporting Info
