# Supplementary material for: Molecular characterization of HLA class II binding to the LAG‐3 T cell co‐inhibitory receptor
Source: Eur J Immunol. 2020 Oct 9;51(2):331–41. doi: 10.1002/eji.202048753 (PMC8101287; doi:10.1002/eji.202048753)
Supplement: Supplementary file 1 — Supporting Info [file EJI-51-331-s001.pdf]

Supporting information Figures S1 –S5

Molecular characterisation of HLA class II binding to the LAG-3 T cell co-inhibitory receptor

Authors and Affiliations

Bruce J. MacLachlan[1], Georgina H. Mason[1], Alexander Greenshields-Watson[1], Frederic Triebel[2], Awen Gallimore[1], David K. Cole[1*] and Andrew Godkin[1*]

[1]Division of Infection & Immunity, Cardiff University, CF14 4XN, UK.

[2]Immutep S.A.S., 91893 Orsay, France.

*These authors contributed equally to the study.

To whom correspondence should be addressed: Professor Andrew Godkin, Email: godkinaj@cardiff.ac.uk, and Dr David K. Cole, E-mail: coledk@cardiff.ac.uk.

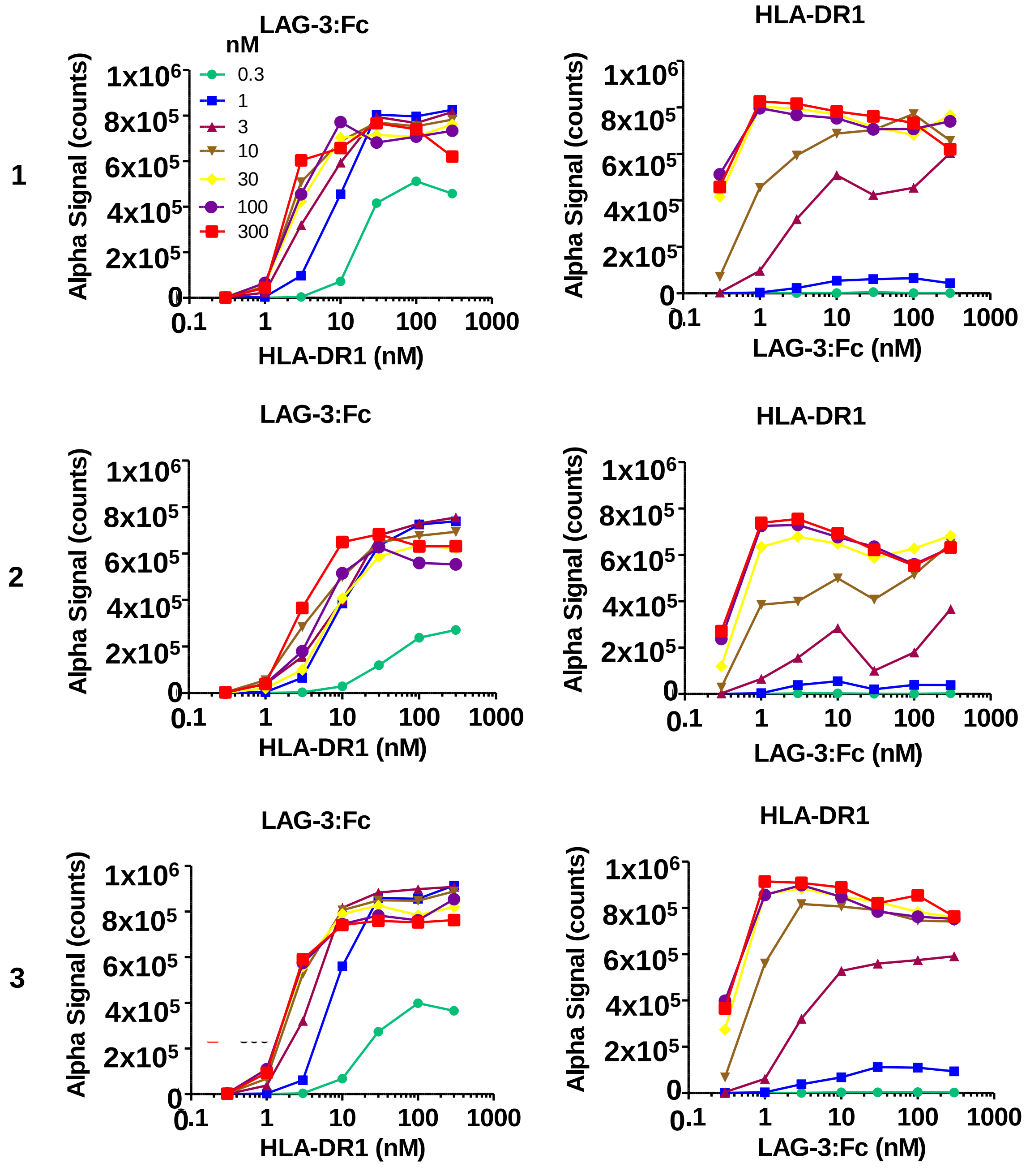

Supplementary Figure S1 – LAG-3:Fc binding detected to multiple pHLA-II alleles. Three replicate protein cross titrations (marked 1-3) using LAG-3:Fc and pHLA-DR1 using a 1 in 3 dilution series from 300 nM to 0.3 nM. All assays were performed using Protein A acceptor beads and streptavidin donor beads at a final bead concentration of 20 µg/mL, in 96 well ½ area opti plates and analysed using an En-vision plate reader at RT.

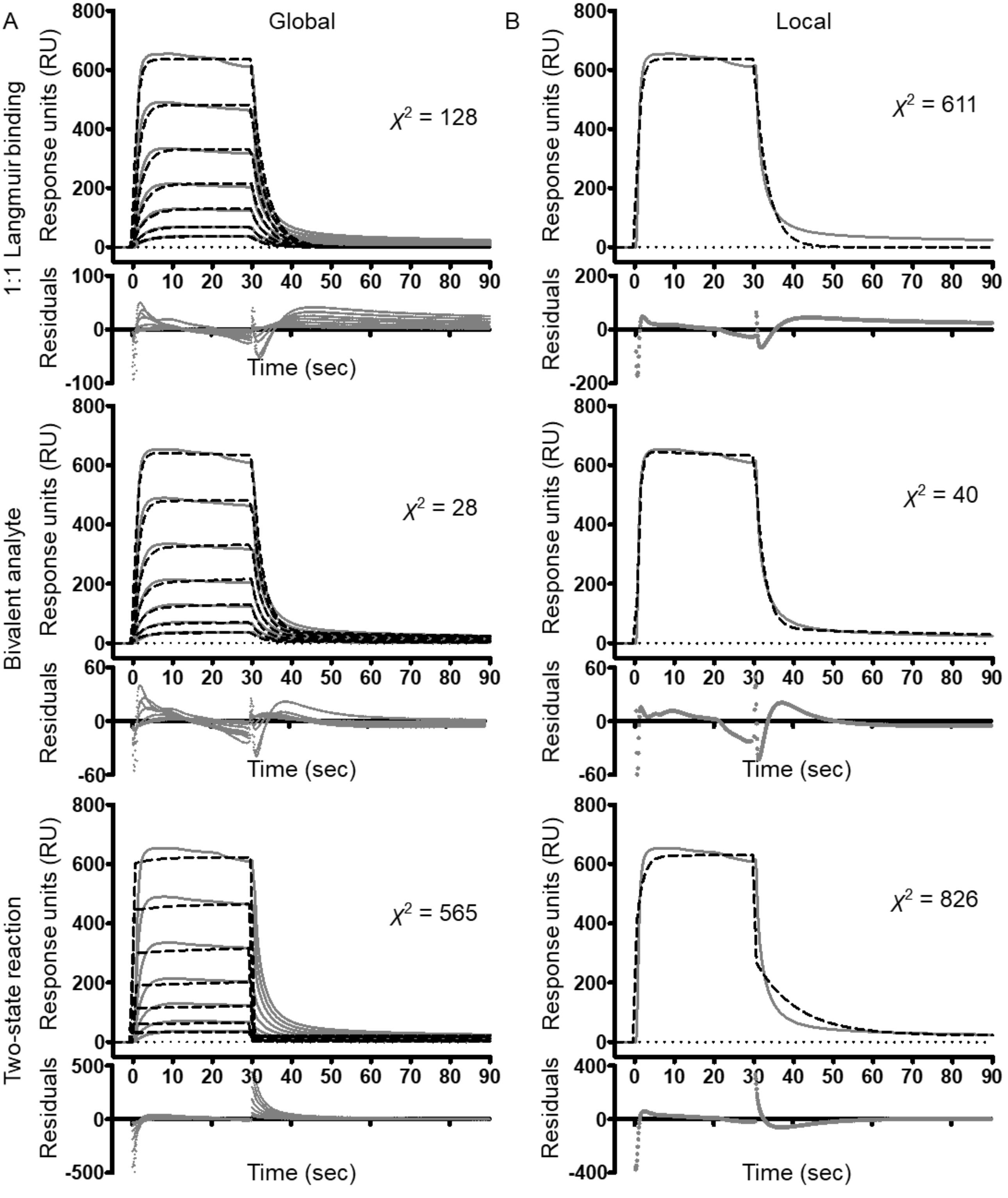

Supplementary Figure S2 – LAG-3:Fc binding to HLA-DR1 exhibits bivalent analyte kinetics.

A) Global fit analysis of reference subtracted sensograms of LAG-3:Fc (0.1 – 7 µM) binding to immobilised (525 RU) HLA-DR1 fitted with three candidate models: 1:1 Langmuir binding, bivalent analyte and two-state reaction. Observed sensograms are shown as grey solid lines, fitted curves as black dashed lines with inset χ^2 value and kinetic derived dissociation affinity constant K_D. Corresponding curve fit residual plots are shown below each fit. Data are representative of three independent experimental repeats. **B)** Local fit analysis of the reference subtracted sensogram describing LAG-3:Fc binding at 7 µM to immobilised HLA-DR1. Data are representative of three independent experimental repeats.

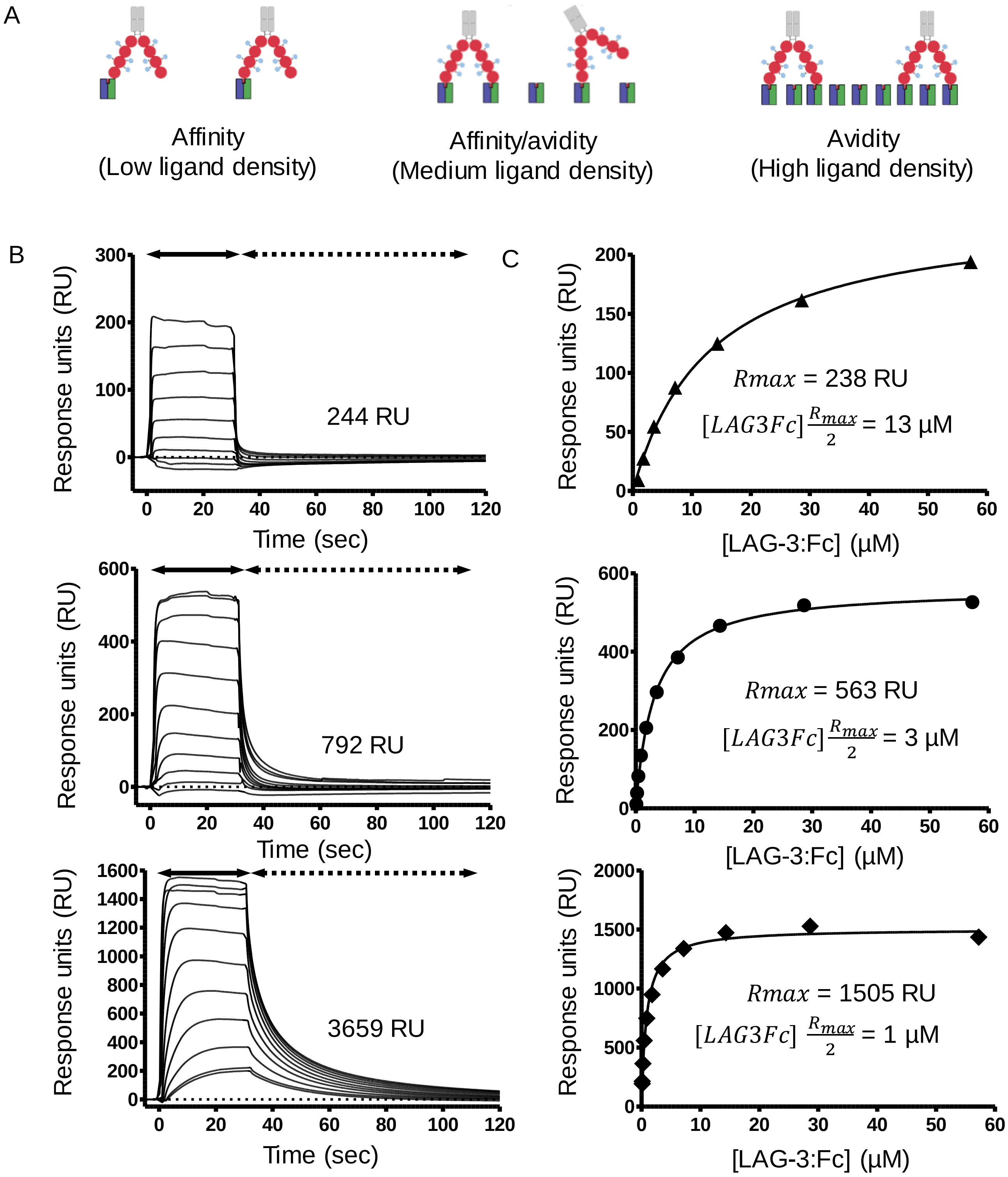

Supplementary Figure S3– LAG-3 binds HLA-DR1 with micromolar affinity. A) Schematic overview of the effects of increasing concentration of ligand on the observed binding of LAG-3:Fc in SPR experiments. **B)** SPR analysis of LAG-3:Fc binding to HLA-DR1 immobilised at low (244 RU), intermediate (792 RU) and high (3659 RU) ligand concentrations. **C)** Steady-state analysis of LAG-3:Fc binding to HLA-DR1 at varying ligand concentrations analysed from sensograms as shown in **A** by plotting RU increase from baseline during steady-state (25 seconds into injections) against concentration of LAG-3:Fc. Data are representative of three independent experimental repeats.

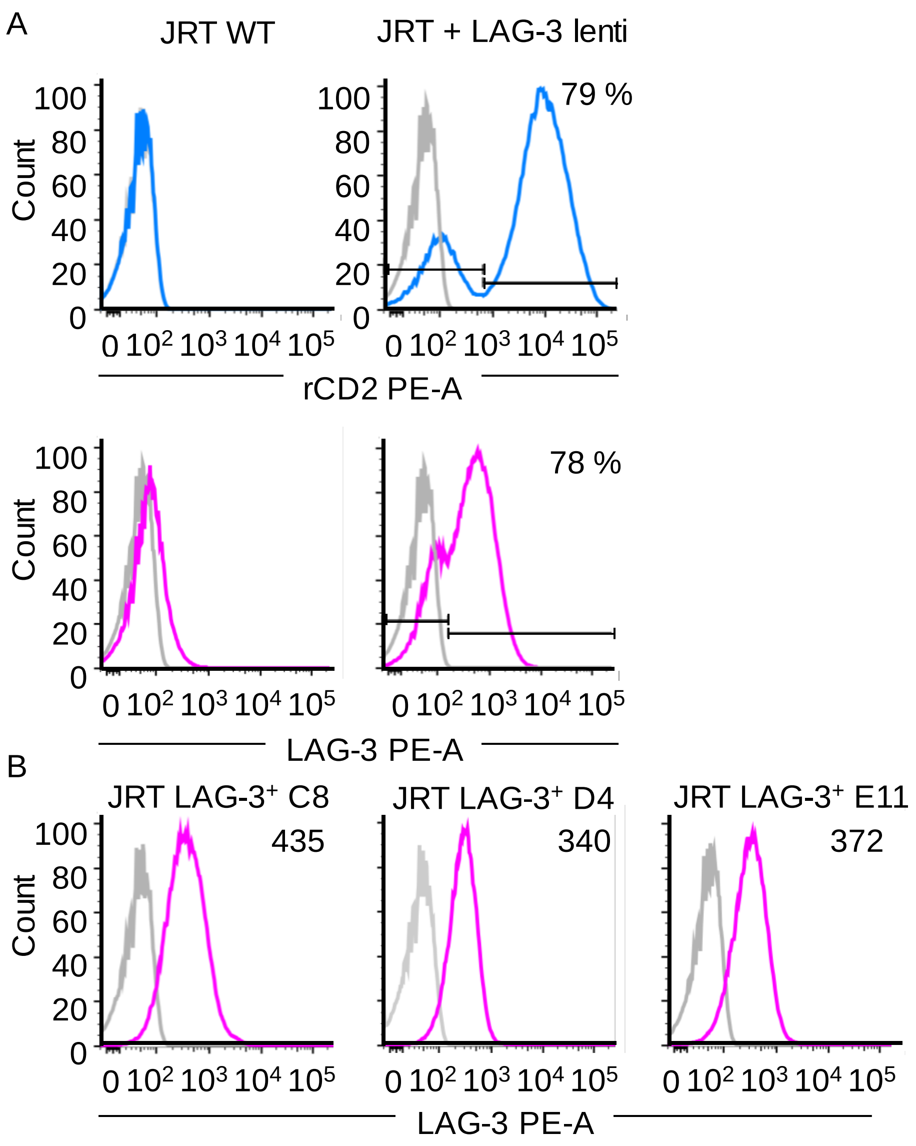

Supplementary Figure S4 – Formation of the JRT LAG-3⁺ C8 cell line via lentiviral delivery. A) Flow cytometry histogram plots of rCD2 and LAG-3 expression in wild type (WT) JRT cells and transduced JRT line following lentiviral delivery (JRT + LAG-3 lenti). rCD2 staining coloured blue, unstained control coloured grey and LAG-3 staining coloured pink. Data are representative of three independent experimental repeats. **B)** Flow cytometry histogram plots of LAG-3 expression in three consequent JRT LAG-3⁺ clones C8, D4 & E11 generated from the line described in **A**. Unstained control coloured grey and LAG-3 staining coloured pink. Inset numbers represent gMFI of LAG-3 staining. Data are representative of three independent experimental repeats.

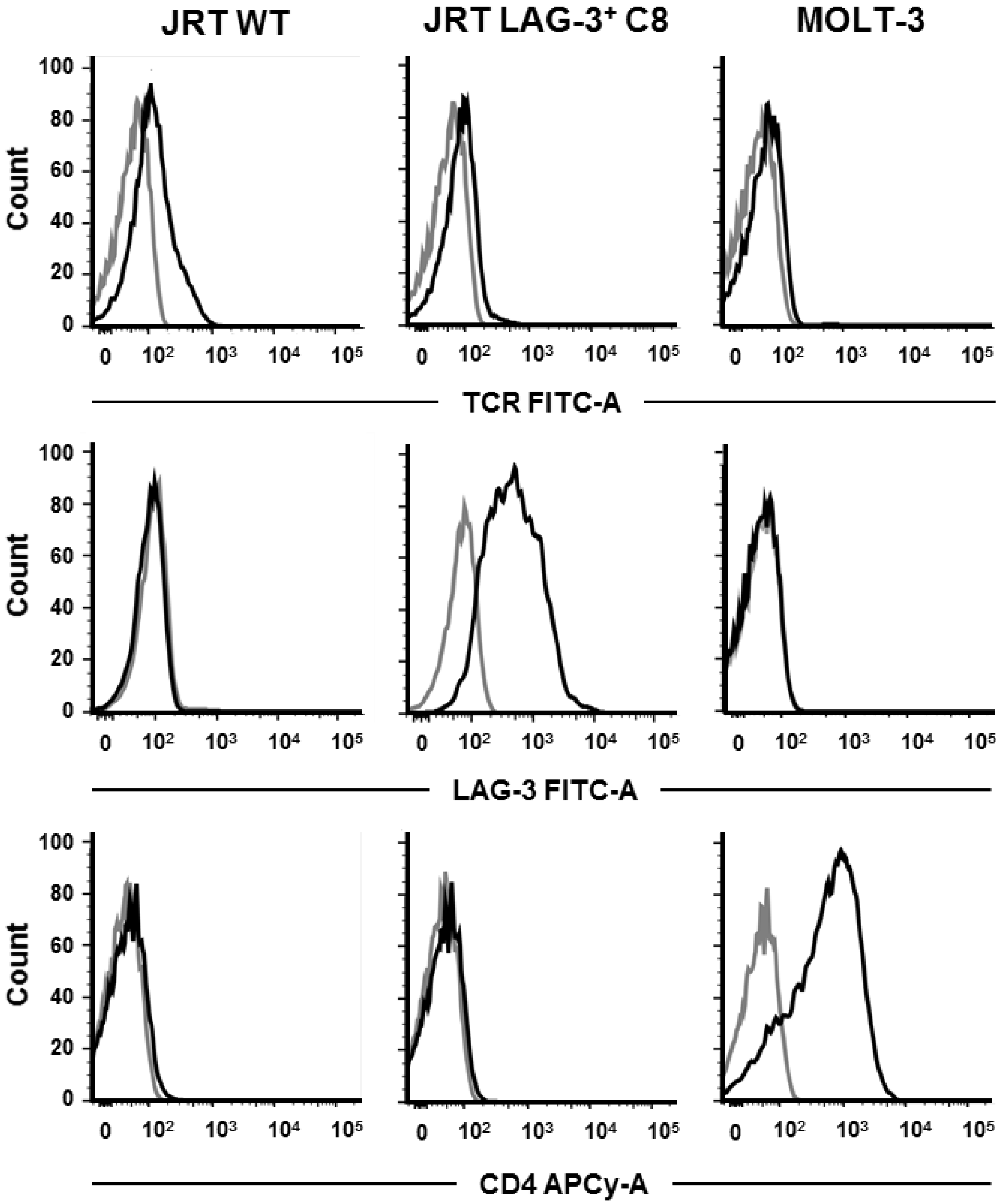

Supplementary Figure S5 – Surface characterisation of T cell derived cell lines. Flow cytometry histograms of TCR, LAG-3 and CD4 surface expression on LAG-3- JRT WT, JRT LAG-3+ C8 and MOLT-3 cells. Stained marker = black, FMO/unstained control = grey. Data are representative of three independent experimental repeats.
